# Opening windows and closing gaps: a case analysis of Canada’s 2009 tobacco additives ban and its policy lessons

**DOI:** 10.1186/s12889-018-6157-3

**Published:** 2018-11-28

**Authors:** Raphael Lencucha, Arne Ruckert, Ronald Labonte, Jeffrey Drope

**Affiliations:** 1 Faculty of Medicine, School of Physical and Occupational Therapy, McGill University, 3630 Promenade Sir William Osler, Montreal, QC H3G 1Y5 Canada; 2 School of Epidemiology and Public Health, Faculty of Medicine, University of Ottawa, Ottawa, ON Canada; 3 Economic and Health Policy Research, American Cancer Society, Atlanta, GA USA

## Abstract

**Background:**

In 2009, Canada adopted legislation (Bill C-32) restricting the sale of flavoured tobacco products, one of the first in the world. This study examines the agenda-setting process leading to the adoption of Bill C-32.

**Methods:**

This research was conducted using a case study design informed by Kingdon’s Multiple Streams framework and Heclo’s policy learning approach. In-depth interviews were conducted with key informants from government, health-based non-governmental organizations (NGOs), trade associations and the cigar manufacturing sector (*n* = 11). Public documents produced by media (*n* = 19), government (n = 11), NGOs (*n* = 15), as well as technical reports (*n* = 8) and formal stakeholder submissions (*n* = 137) were included for analysis. Data were coded with the objective of understanding key events or moments in the lead up to the adoption of Bill C-32 and the actors and arguments in support of and opposition to Bill C-32.

**Results:**

The findings point to the importance of a small but active group of NGOs who worked to publicize the issue and eventually take advantage of an open policy window. Our analysis also illustrates that even though consensus was developed about the policy problem and civil society was able to garner political support to address the problem, disagreement and dissent pertaining to the technical dimensions of the policy solution created loopholes for the tobacco industry to exploit.

**Conclusions:**

NGOs remain a critical factor in efforts to strengthen tobacco control policy. These organizations were able to mobilize support for the tobacco flavouring ban adopted at the Federal level in Canada, and although the initial Bill had major limitations to achieving the health objectives, the persistence of these NGOs resulted in amendments to close these loopholes.

## Background

“Tobacco companies are using candy-like flavors and high tech delivery devices to turn a blowtorch into a flavored popsicle, misleading millions of youngsters to try a deadly product” [1].

Tobacco remains a leading health threat of our time, with more than 7 million smoking-related deaths globally each year [2]. Although many lessons have been learned about establishing comprehensive and effective tobacco control policy, the recent rise of flavoured tobacco products and government efforts to regulate such products remain understudied. Tobacco companies have invested heavily in new tobacco products with appealing flavours [3], raising concerns about how such products might attract youth and entice a new generation of tobacco users. As a result, some countries are pursuing or exploring policy on flavoured tobacco products, including one of the more popular categories, menthol cigarettes. This raises questions about effective strategies and inherent challenges in regulating such products.

Previous scholarship has identified several key ingredients to moving issues onto the tobacco control policy agenda, including: non-governmental organization (NGO) lobbying [4, 5], a robust and policy-targeted evidence base [6], policy champions within government pushing the policy agenda forward [7], and, more recently, the Framework Convention on Tobacco Control (WHO FCTC), which appears to have led some states to strengthen their national tobacco control policy [8–10]. There also exists a good understanding of the barriers to effective tobacco control policy, largely centered on the actions of tobacco interests in mobilizing resistance to tobacco control policy at different levels of government [11, 12] and in different international venues such as the World Trade Organization [13, 14]. Another recent corporate strategy, of particular importance to this research but not as widely discussed in the academic literature, consists of finding loopholes to circumvent new tobacco regulations, for example through creation of new product classes (that are similar or almost identical to banned products) but are not covered by existing regulations [15, 16].

Canada has been viewed as a model for tobacco control policy since it began passing tobacco control legislation in the late 1980s. Along with the US Family Smoking Prevention and Tobacco Control Act, and subsequent FDA regulations, Bill C-32, An Act to Amend the Tobacco Act, was the first legislation in the world to control flavoured tobacco products. This tobacco control measure provides an important case to examine the factors and circumstances that helped propel flavoured tobacco products onto the policy agenda and ultimately led to the adoption of legislation to control these products. The purpose of this study is to examine the factors that led to the adoption of Canada’s first tobacco additives ban (Bill C-32) in 2009. This study demonstrates that legislative and regulatory successes remain hard won, are inextricably linked to the efforts of policy entrepreneurs from the NGO sector, and that even when successfully adopted, novel regulations may face technical loopholes unanticipated by legislators and/or regulators. Drawing lessons from the Canadian experience is particularly important as other countries navigate new terrain in tobacco control, such as regulating flavoured tobacco, plain and standardized packaging, and electronic cigarettes. Examination of the agenda-setting process also contributes important lessons for countries attempting to implement the provisions of the WHO FCTC, including but not limited to Articles 9 and 10, which address tobacco additives.

### Theoretical approach

We draw on Kingdon’s multiple streams framework to help explain the establishment and eventual adoption of Bill C-32. Kingdon’s approach has been applied to the study of tobacco control [17, 18] and provides the conceptual framework for our data analysis. Kingdon argues that policy change occurs when there is adequate attention to a problem (the problem stream), a policy solution has been clearly articulated and reached consensus (the policy stream), and there is political will to adopt this policy (the politics stream). When all three streams converge, a policy window opens, representing “an opportunity for advocates of proposals to … push attention to their special problems” [19]. Kingdon suggests that windows can be opened by external focusing events, such as crises or accidents, or by institutionalized events, for example elections. Policy entrepreneurs play a central role in shaping the course of the three streams and their intersection by linking policy problems and policy solutions with political opportunities [20].

Kingdon’s framework is most helpful for the adoption of legislation, but to understand subsequent implementation, it is necessary to complement it theoretically. Accordingly, we borrow from Heclo’s conceptualization of the development of welfare policy in Great Britain and Sweden [21], an approach that was subsequently refined by Sabatier [22] and many others. Heclo posits that policy-oriented learning occurs among key stakeholders in the coalition of actors promoting a broader policy effort. In this understanding of the policy process, actors, partly through trying new policy approaches, learn from their mistakes and successes and adjust their approach and policies accordingly.

## Methods

This research was conducted using a case study design. This design focuses on a bounded policy context in order to systematically examine the process surrounding the policy [23, 24]. In-depth semi-structured qualitative interviews were conducted with key informants between July and October 2014 (*n* = 11) (See Table 1). Key informants were recruited based on their known involvement in the legislative process leading to the adoption of Bill C-32. Informants were recruited from government, health-based NGOs, the tobacco industry (cigar manufacturers), and trade associations. Snowball sampling was used to recruit participants who were suggested by other participants [25]. Our aim in these interviews was to gain a rich understanding of the policy process as well as key issues associated with the policy content. Interestingly, there was remarkable consistency across all 11 informants in their recounting of the key events or moments that occurred in the lead up to the adoption of Bill C-32 and in the subsequent implementation. All interviews were recorded and transcribed verbatim.

**Table 1:** Key informants

Participant number	Category	Interview length
1	Trade association	56:02
2	Government	40:34
3	Cigar manufacturer	83:26
4	CSO	61:34
5	CSO	64:18
6	Government	32:00 (not recorded)
7	Independent consultant	21:12
8–9	CSO	66:10
10	CSO	47:35
11	CSO	30:00

Public documents related to the proposed legislation were also included for analysis, consisting of media accounts (*n* = 19), government reports (*n* = 11), NGO briefs (*n* = 15), technical reports prepared as part of the legislative process (*n* = 8), and formal stakeholder submissions on the proposed Bill (*n* = 137). All public documents were identified using online searches. The documents were used to provide a broader perspective on the arguments and events that were taking place prior to the adoption of Bill C-32. These documents were used to triangulate the findings from the interviews with key informants. The stakeholder submissions were acquired through a freedom of information request and contain all submissions on file. The documents were systematically analyzed and grouped based on key messages presented both in favor of and in opposition to the proposed policy measures. We also collated the recommended amendments presented throughout the process. All data were organized and analyzed using NVivo qualitative software. We used open coding to understand stakeholders’ responses and actions in support of, and opposition to, Bill C-32. The data were coded according to the key events or moments in the policy process, arguments for and against the Bill and challenges and opportunities that occurred during the policy process. This coding process involved looking for commonalities and differences in the informants’ accounts of the process leading to the adoption of Bill C-32. Kingdon’s Multiple Streams theory as well as Heclo’s policy learning theory on policy process informed this data analysis. The lead author conducted the initial analysis. AR conducted a second analysis of the transcripts. The two authors compared findings and any differing interpretations were resolved through discussion. However, there was general agreement about the key events identified, the thematic structure of the arguments for and against the Bill and key challenges and opportunities identified by the informants. The document analysis was also used to corroborate the results from the interview analysis. Ethics approval was granted by the Institutional Review Board of McGill University.

## Results

We begin our analysis by discussing how the issues were framed and presented as a problem to be addressed by policy. We then present how the problem stream converged with the political stream when a policy window was opened. We conclude by analyzing the factors that contributed to the limitations of the 2009 legislation and subsequent amendment.

### Problem identification and framing—the problem stream

In 2008, statistics on tobacco use in Canada showed that cigarillo sales jumped from less than 50,000 units in 2001 to more than 80 million units in 2006 – the period when colourful, flavoured products entered the market [26]. At the same time, the Canadian Tobacco Use Monitoring Survey was released with a corresponding report on cigarillo smoking in Canada. The results indicated a rise in flavoured tobacco consumption among youth, finding that one third of youth aged 15–19 had tried these products. The report was used by advocates as a basis for generating momentum towards banning tobacco additives. The concern was that flavoured tobacco products might be a new corporate strategy to encourage teens to initiate tobacco consumption. One study participant (P5), an early advocate for a ban on flavoured products, noted that the survey “show[ed] the very significant youth use of cigarillos” providing the ammunition needed to draw attention to the issue. Another participant pointedly expressed that “we wanted to see a ban on flavored additives being used in tobacco” (P10), with a particular emphasis on the youth demographic that consumed these products. Of particular concern was the finding that “we saw smoking rates declining amongst cigarette users in high school students (but) we saw this kind of sharp increase in the use of cigarillos” (P10). These data allowed NGO actors to frame the issue of flavoured tobacco consumption through the lens of youth protection. At the same time a major youth-led public campaign was launched to bring the topic to the public’s attention. The campaign was entitled “Flavour … Gone!” and it was strategically supported by some larger NGOs and further reinforced youth framing of the issue. As one of the Flavour … Gone! co-organizers noted: “I don’t think anyone who sees the flavoured tobacco products like cigarillos and chew tobacco that are on the shelves of Canadian stores can fail to see that they would be especially attractive to youth” [26].

Despite an emerging public health consensus about the urgency of the problem, several informants noted that little initial attention was being paid to the issue by the wider NGO community or government agencies: “Health Canada officials were ignoring them (flavoured tobacco products).” (P8–9). A small number of NGOs were eventually able to enlist the support of a wider group of NGOs working in tobacco control and youth protection, creating a stronger consensus base to address the issue. As one participant highlighted, “There was a lot of cohesion. … I think Physicians for a Smoke Free Canada certainly played a role in creating that cohesion but the issue itself just had consensus…. Everybody was working together” (P5) and that “Physicians for a Smoke Free Canada, Canadian Cancer Society, Quebec Coalition for Tobacco Control were among the key supporters, but there were very many other health groups as well” (P10). These three NGOs have been a part of the fabric of tobacco control in Canada over the past 40 years^1^ and played a prominent role in strengthening national and subnational tobacco control measures, as well as contributing to the establishment of the WHO FCTC [27, 28].

### Emerging policy consensus—the policy stream

For Kingdon, the policy stream is where solutions are generated to address a particular problem [19]. The health NGOs, in addition to identifying and garnering support to address the broader problem of increasing youth consumption, were also generating tangible policy solutions, including calls to restrict the ability of tobacco manufacturers to add flavouring agents to tobacco products; ensure health warnings on all products containing tobacco; increased penalties for the sale of single cigars and blunt wraps (i.e. tobacco leaf wrapper) to youth; regulating packaging size of cigar and blunt wraps; and ensuring that small cigars could not be sold individually [29]. These policy solutions were partially incorporated in a Private Members Bill, which focused on banning all flavouring agents in tobacco products, ensuring that cigarettes or cigarillos are sold in packages that contained at least 20 units, and that all packages of pipe tobacco or cigars displayed health warning labels occupying at least 50% of the panel [30]. This first uptake at the policy level was from a Member of Parliament for the New Democratic Party, Judy Wasyliycia-Leis, who, with the support of one of the NGOs working at the federal level, introduced the Private Members Bill into Parliament in June 2008 (Bill C-566 “An act to amend the Tobacco Act”). Although the Bill only made it to first reading and did not receive royal assent, one of the participants noted that “The first reading of the Bill C-32 I think came close to the point where private member’s bill was getting for discussion. The arguments were as they are today… look at the sales data, look at the youth use…they should be banned. It makes no sense for these things to look like (candy)” (P10). Those advocating for the ban on flavoured products initially emphasized the need to ban flavours in combustible and smokeless tobacco products without much discussion of electronic nicotine products, which had not yet emerged on the market as a serious concern. It is noteworthy that the initial policy formulation did not consider the weight of the product and how this weight was to be used to differentiate between products included in the ban. Weight would eventually serve as a major limitation of the legislation.

### Flavoured tobacco legislation in Canada—the politics stream

The politics stream refers to the political context and specifically the conditions that lead to receptivity of those with power to decide on policy solutions to address an identified problem. Even though the private members bill (Bill C-566) was voted down in Parliament, it drew attention to the issue of tobacco additives for the first time inside Parliament. Apart from widespread recognition that this policy problem required a technical policy solution, we found that it was the strategic appeal of the issue in the politics stream that pushed this issue onto the agenda. All participants in this study independently identified one event as *the* “turning point” (P4, P7) that led to the establishment and eventual adoption of Bill C-32. The event itself was an upcoming federal election in which the Conservative Prime Minister, Stephen Harper, was preparing to run for re-election. One key informant involved in Harper’s campaign for re-election noted:

*It was a lot of good timing in a lot of ways because you know we just happened to be at this meeting trying to push this issue during a federal election, where the government wanted to reach out to the same people these products were being targeted to.* (P7)

The “turning point” arrived when representatives from one of the national NGOs were invited to a meeting with members of a national medical organization. At this meeting the representatives from the NGO were advocating for support to tackle the issue of flavoured tobacco products and had brought samples of the colourful packages in which the products were being sold. Two of these representatives noted that:

*We had lunch and one of their people (from the medical organization) comes in, then their public relation person takes the products and goes to show their communication director (name) who was previously (involved in the election campaign of) Stephen Harper [former Prime Minister of Canada]. During the election campaign, he went to the Conservatives and said, “look it’s an easy slam-dunk thing”. So, the policy was announced during an election campaign by Stephen Harper at someone’s kitchen with a bunch of kids around. … a quick media spell, quick issue to throw into the campaign.* (P8–9)

One of the participants involved in this encounter recounts that the product packaging had triggered his interest in the issue, providing him with an opportunity to inject the issue into the election campaign:

*And when they took out the packages it was a series of very slick, glossy products like in a variety of flavours; tangerine, peanut butter. They were a combination of small cigars and blunts that were, in my mind anyways, indistinguishable from fruit roll-ups you would put in your child’s lunch. … So, they (campaign team) said that’s terrific… that’s terrible, but it’s terrific as far as this campaign goes because we are going to be in Western Canada in the next week, we are going to be doing an announcement at a kitchen table directed at families and you know we were not sure what the announcement was going to be, but it is now going to be this.* (P7)

From this encounter, the issue was worked into the schedule for the campaign announcement in less than 48 h, and “the fact that it was the election promise made by the Prime Minister on the campaign trail guaranteed that it would go through and … go through quickly because it was all about keeping promises” (P7). This strategy was confirmed in an example provide by an NGO representative to the Standing Committee on Health where he presented similar products to the Committee noting “As one example, I have these Bravo cigarillos that are packed to look like magic markers or lip gloss. The Prime Minister held these up during his announcement. I’ll pass these around to members of the committee.” (Rob Cunningham, 31). Another NGO representative used the same strategy at the Committee meeting: “I see that we’ve all brought a lot of samples for show and tell, but I’m going to pass around a few cigarillos, and I invite you to just open the cap and smell them. They really do smell like candy or Kool-Aid and nothing like a tobacco product.” (Melodie Tilson, [31]).

This story captures three important aspects of the process leading to the adoption of Bill C-32. First, the NGO representatives had already been working to move the issue of flavoured tobacco to the federal policy level and had the strategic foresight to bring samples of the product packages to the national medical organization meeting. Although the opening of this policy window at this particular moment was a critical turning point in the lobbying efforts, it is important to emphasize that this encounter was one of many that had been actively pursued by NGO representatives over a number of years. The persistent and systematic pursuit of legislation to address flavoured tobacco products by a small group of NGOs with a history of successfully pushing for stronger tobacco control in Canada is the back story to the more dramatic and acute encounter described above. As noted, the informants confirmed that the visual and tangible demonstration of the products at the center of this issue were important to capture the attention of those with power to develop policy. Second, the timing and graphic nature of the products were coupled with the “youth protection” frame to enhance the salience of the issue during the meeting. Third, this framing aligned with the political strategy being crafted by the Prime Minister’s re-election campaign team and continued to be used by those supporting Bill C-32. Of the 137 public submissions to the Senate Standing Committee, 98 highlighted the importance of protecting youth as the policy goal. The “youth” frame was strategically appealing to the ruling political party which needed an issue to enlist support from a particular demographic (i.e. concerned parents). However, having the problem converge with the political stream did not ensure that a comprehensive (or completely effective) policy solution was ultimately adopted.

### The limitations of bill C-32: from political leaps to policy loopholes

Bill C-32 was criticized by tobacco control proponents for only including combustible tobacco products and for permitting the continued sale of menthol flavoured tobacco. Beginning with the first meeting of the Senate Standing Committee on Health to discuss the Bill, NGO representatives pushed for the inclusion of both smokeless tobacco products and to reverse the exemption of menthol in the ban. One representative noted “Also, in terms of chewing tobacco, from my personal experience, I can say that the difference between regular Skoal (chewing tobacco) and any kind of flavoured Skoal product is night and day. Most users, when they first use a straight product—a Skoal straight product—will puke, whereas the other products are quite palatable.” (Sam McKibbon, 31). Another NGO representative expressed the need to include menthol products in the ban: “Our second amendment is with respect to the menthol exemption. The government’s intent is to maintain an exception for menthol cigarettes, but it still would be possible to ban menthol little cigars, menthol smokeless tobacco, and menthol blunt wraps. We propose an amendment to ban menthol from those other product categories, not touching the government’s intent.” (Rob Cunningham, 31). Although these suggestions were not taken up as amendments to the Bill, there was wide recognition amongst our study participants that even though the Bill was not comprehensive, it represented important progress for tobacco control in Canada. To confirm this impression, a recent study by Chaiton and colleagues [32] did find that the volume of flavoured cigar sales decreased following the adoption of Bill C-32 in 2009, while also confirming that the absence of a menthol ban did result in an increase in sales of menthol products. Despite the mixed success of Bill C-32, the NGOs who had been advocating for the legislation viewed Bill C-32 as an incremental step towards the inclusion of all flavoured tobacco products in a more comprehensive ban.

In what is a crucial policy lesson, however, we found that none of the health advocates or government officials in favour of controlling flavoured products had anticipated that the weight threshold in the Bill would have such wide-reaching implications for the success of the legislation. The technical oversight was the chosen threshold of 1.4 g or less to define included products. One informant noted that “It was literally just a matter of weeks before companies … drove trucks through the loophole.” (P8–9). Another informant noted that “As soon as the new regulations came into effect in July 2010 new flavoured tobacco products that are marginally bigger than 1.4 grams appeared on the market” (P4). This was noted by another informant from government who stated:

*It did not actually meet the objective of the Bill which was to keep these products, which are candy flavoured tobacco as a, whatever you call it, entry level tobacco, out of the hands of kids. They [tobacco companies] just made it bigger.* (P2)

The cigar manufacturers had voiced opposition to the 1.4 g threshold during the public consultations and one informant, a cigar producer, noted explicitly that “the loophole is actually the weight” (P3). This participant viewed the products that the Bill was targeting (cheaper cigarillos which were being sold individually enhancing their affordability to youth) as distinct from those which his company produced (full size cigars that were much more expensive). Some informants attributed this oversight to the fact that the Bill was one of the first attempts in the world to control flavoured tobacco products:

*You know, this is a ground-breaking legislation. No other country had banned flavoured cigarillos. In hindsight the 1.4 g threshold is totally inadequate but at the time it was very significant and important.* (P10)

Although our informants were unable to identify the origins of the decision to implement a 1.4 g threshold, we were able to trace this weight to the United States Department of Agriculture definition of “small” cigar by the weight of 1.36 g. The cigar producers had also correctly anticipated that the weight threshold was not going to be an effective approach, noting:

*What we did at that point was sort of present the same arguments that there are traditional cigars that weigh less and the weight is not the right approach. Because it’s not the weight ... because there are little cigars that are made in Europe that have been smoked there for 200 years and they are smoked by adults. From a price point in the market place they cost twice as much the pack of cigarettes do. Clearly, if a kid wants tobacco he gets twice as many cigarettes or tobacco products [for the same price as] a traditional European cigar.* (P3)

The ultimate inadequacy around the Bill’s technical details illustrates that these aspects of were far more complicated than what those who were invested in the Bill had anticipated.

The debate about including smokeless tobacco in the Bill was more vigorous. Participants offered different interpretations for why smokeless tobacco was not included in the Bill. However, several informants noted that Health Canada had justified the exclusion of smokeless products by arguing that the rates of smokeless tobacco consumption were low across Canada. One informant expressed that Health Canada was relying on population data in the aggregate, suggesting that this evidence was not capturing the higher concentration of smokeless tobacco use in sub-regions and populations:

*And what Health Canada claims is…well, you know there’s hardly any snuff sold in Canada. (They say) we have our survey data to prove it. Well, the population of North Western Ontario compared to the population of Canada is ….and there are a few other places, Alberta and North West Territories and Interior BC and maybe the Yukon, where the snuff use is more wide-spread than elsewhere. … These are not populations that are going to get picked up in national surveys … They kept saying, oh well, the survey data does not show anything.* (P8–9)

During the consultation process, 104 of the 137 submissions encouraged the government to include smokeless tobacco in the Bill. These submissions followed two themes: first, the disproportionate consumption of smokeless tobacco products by youth when compared to adults; and second, the regional concentration of use in rural and northern Canada, and the prairie provinces. As one individual noted during one of the Standing Health Committee meetings: “Unfortunately, this bill will not ban the use of flavouring in smokeless tobacco. As a result, youth in regions like Northwestern Ontario where smokeless is a growing problem, will not receive the protection they need.” [33]. From the analysis of the public documents, Imperial Tobacco Canada and Brad Rodu, a professor of medicine in Kentucky known to accept money from the tobacco industry, were the two main opponents of including smokeless tobacco in the Bill. The thrust of their argument was that smokeless tobacco is a less harmful alternative to combustible tobacco products, going so far as to state that “The House of Commons does not want to be in the position of banning smokeless tobacco products that have minimal to no adverse health consequences, while at the same time maintaining and promoting the market dominance of cigarettes” [34]. Given that we were not able to obtain information on how the government formulated its decisions on what the Bill would include or not include it is difficult to explain the government’s resistance to including smokeless tobacco in the original Bill. Despite numerous attempts, we were unable to recruit the key informant in Health Canada who led the drafting of Bill C-32.

The key informants who supported the Bill noted that once the issue of regulating flavoured tobacco products was on the agenda of the re-elected government, following various campaign promises, the government seemed reluctant to address the concerns about the content of the policy. Health groups and some provincial governments responded to the omissions by pushing for amendments to the federal legislation, but it took another 5 years until amendments came into force. Amendments did eventually extend the flavouring ban to other types of cigars at the federal level (> 1.4 g to ≤6 g), although with an exception for “traditional alcohol flavours” (port, wine, rum and whisky) [35]. In April 2017, the federal government further amended the Tobacco Act to remove the exception for menthol additives, thus prohibiting their use in cigarettes, blunt wraps, cigarillos, and the types of cigars noted above, effective October 2, 2017. The menthol ban meant that by the end of 2017, 95% of all flavoured tobacco products had been removed from Canada’s tobacco market [36].

The long-run perspective on this case is important. Many of the NGOs who had been advocating for the additives ban in 2007 had noted in the interviews that they recognized that there were limitations in the original Bill C-32 but expressed that having this issue on the agenda and adopted by the Government was an important step. This incremental approach had proven successful to the extent that the amendments have expanded the scope of the legislation to menthol products and addressed the weight loophole. There are examples that suggest this incremental approach is not always successful and that concessions can remain embedded in legislation and regulations with little political will for amendment. For example, the US Family Smoking Prevention and Tobacco Control Act that was signed into law in 2009 excluded menthol products (for largely political reasons given the domestic production and consumption of such products) and many argue that this exception has made it difficult to tackle youth consumption.

## Discussion

Our analysis provides insights into the driving forces behind the adoption of Bill C-32 on flavoured tobacco products, while also highlighting some of the challenges inherent in the Bill as well as the factors that led to the eventual amendment of the legislation. In Kingdon’s framework, the problem stream captures how an issue is framed to define its political image and promote its inclusion on the political agenda [37]. We noted earlier that framing the issue in terms of youth protection was crucial at the stage of generating NGO support, even though there was initially no formal uptake of the issue by the government. Such problem framing nonetheless set the stage for NGOs to take advantage of the opening of a policy window when their efforts converged with the political stream. The importance of framing in agenda setting has been widely acknowledged in other studies on tobacco control, and use of ethical frames (e.g. youth protection) has been argued to be an effective counter-frame to the tobacco industries’ freedom of choice frame (i.e. protector of individual choice) [38]. The focus by the Conservative Canadian administration on protecting mothers and children as part of its electoral campaign provided the needed policy window, a short-term opportunity characterized by greater political receptivity to NGOs’ lobbying for a tobacco flavouring ban. Kingdon contends that policy agendas can change when “policy entrepreneurs” (in our case, the organized group of health NGOs) are ready to capitalize on these open windows with a captivating policy problem and proposed solutions [19] (only possible due to their earlier work leading to a consensus on the policy framing and regulatory options).

The way that this issue entered the political agenda is an important counterpoint to many rational or evidence-based conceptualizations of the policy process. Although the NGOs were drawing from evidence of youth consumption of flavoured tobacco products, this evidence alone was not sufficient to attract the attention of political authorities. As one participant suggested, decisions-makers had become somewhat immune to new evidence on tobacco harms. In the case of Bill C-32, tobacco control writ large did not itself appeal to decision makers as a salient policy goal. What drew attention to the issue was the presentation of the product packages combined with the youth protection frame. Put another way, the trigger was an emotional response tied to a strategic opportunity. This finding supports research by Studlar, who argues that “although tobacco control is an issue dependent on science, in the public debate, increasingly technical dimensions have declined in favor of more readily understood ones, including the morality of manufacturing products injurious to public health” [5].

This policy problem-political salience dynamic also sheds light on how political interests can sometimes override commercial interests. There is a foundation of empirical research on tobacco control that documents systematically industry manipulation in the public sphere. In this case however, the political stream aligned with the policy and problem streams in a way that allowed the agenda to be set in favour of resolving a novel tobacco control problem. This finding corresponds with numerous recent studies examining the political factors that facilitate public health policy formation and agenda setting ranging from taxation on sugar-sweetened beverages [39], cervical cancer screening and treatment [40], health care reform [41–43], and HPV screening and vaccination [44, 45]. Having the problem stream converge with the political stream in this case did not ensure that an appropriate policy solution was developed, legislated, and implemented. As several of our informants noted, there was resistance to addressing the quickly apparent loopholes in the policy. Our study, however, was unable to determine what was driving this resistance amongst legislators.

One explanation may reside in party affiliation—and perhaps associated ideologies— being associated with divergent degrees of support for tobacco control measures in Canada. Conservative party members are least likely to embrace or follow through with tobacco control measures, as compared to Liberal, or progressive political parties, such as the New Democratic Party in Canada [46]. Conservative party members have also been shown to be less supportive of tobacco control measures [47], especially if they interfere with basic individual choices, such as limiting what flavour of tobacco one might choose [46]. Research on the legislative process in the United States - at least at the state/sub-national level - has observed similar patterns wherein Republican state legislators are less likely to support tobacco control legislation [48]. This has been attributed to a mix of personal/political norms (freedom of choice), an overall anti-regulation bias, and a lack of knowledge about the harmful effects of smoking. This dynamic might explain why the Conservative administration was unwilling to address the loophole in the Bill after it became obvious that the loophole did indeed exist. Only during the next election campaign (in 2015) did the issue re-enter the political landscape, with the amendment of Bill C-32 to close the loophole. Although we cannot claim with certainty, this pause in political attention to the issue suggests that tobacco control policy was used strategically by the Conservative administration to further its own political agenda, rather than principally from a concern with tobacco control, or even youth protection, more generally.

Finally, there are some important limitations of Kingdon’s multiple streams framework and therefore of our analysis. One shortcoming is that the framework stops short of capturing the full process of how an idea eventually becomes implemented as an effective policy, instead focusing mostly on agenda-setting and policy adoption. Even after an issue appears on the policy agenda and garners wide-spread support in the political system, there are many more obstacles before a proposal becomes law; and even after that, there are potential barriers to the implementation of the law into the envisioned policy [29]. In our case, not closing a regulatory loophole that allowed tobacco companies to develop slightly larger products undermined the intention of Bill C-32. Kingdon has little to say about the (often corporate) forces that push back once an issue has been included on the legislative agenda, and overall does not adequately acknowledge the influence of the industry in shaping legislation and regulation by successfully affecting the decision making of both the legislators and regulators, advancing widespread challenges in tobacco control specifically [49], and ‘chilling’ public health regulation more generally [50]. While loopholes exist because they may often be difficult to foresee, they can also endure as corporate lobbying and influence on the political process can create strong opposition to their closure [51]. The case of the 1.4 g weight threshold in Bill C-32 thus serves as an important reminder of the technical dimensions that remain in tobacco control policy. It also reflects a need for policy learning among both advocates and tobacco control proponents within government. Particularly important in this case was the learning and subsequent advocacy by NGOs who were able to extend their advocacy beyond short political cycles to effect change across administrations. At the same time, the optimistic message for incrementalism is that although it took almost a decade for the Bill to be amended, the policy remained on the advocacy agenda. The issue was kept on the agenda by the same persistent NGOs at the federal level that pushed for Bill C-32 in prior to its adoption and pressure from the provincial governments who had passed provincial legislation banning menthol and other products not included in Bill C-32 [15]. Nonetheless, having the Bill adopted in 2009 was seen as an important step towards stronger tobacco control policy in Canada, which supports the idea that policy agenda-setting via the various convergent elements articulated in our analysis is only part of the story. One could say that the technical limitations of Bill C-32 were addressed through a process that was driven by NGOs who were monitoring the effectiveness of the Bill and who continued to identify and propose solutions to address its technical loopholes. In this sense, Heclo’s policy learning approach sheds important light onto the problem of policy process when incremental victories are followed by persistent remedies [21, 52].

## Conclusions

We highlight two key study findings on the agenda-setting process for tobacco control in Canada. The first underscores the importance of having policy champions, in this case advocates for regulations on flavoured tobacco additives at the federal government level. It was the existence and persistence of a small but active group of NGOs that decided to push tobacco additives as a novel policy problem that required a federal policy response and led to creation of a broader coalition of NGOs. Canadian NGOs have long been active in building relationships with the federal government and are seen as a credible source of policy innovation in the tobacco control area [27, 28, 53] and have historically received direct funding from the government [54]. This case study supports other arguments on the need to build active and longstanding civil society organizations in each country to engage governments (particularly when policy windows open) to adopt stronger tobacco control measures [4, 55], and global health initiatives more generally [56].

The second key finding is an important lesson for other countries following Canada in regulating flavoured tobacco products: even when political actors agree on a policy problem and identify solutions to that problem, tobacco companies will attempt to circumvent regulations by exploiting regulatory loopholes. Additionally, governments often craft legislation that attempts to portray concern for a societal problem while deferring to corporate or other pressures that weaken the effect of the legislation. This challenge is amplified when those advocating for strong legislation do not foresee technical limitations in the legislation, as was the case with Bill C-32. This means that the technical dimensions of proposed policy solutions need to be carefully assessed to make circumvention less likely. This necessarily should include learning from the experiences of early adopters of tobacco control policies (such as Canada’s) to reduce the likelihood of repeating such loopholes. To highlight just one recent example, even before the clove cigarette sales ban went into effect in the US following the Family Smoking Prevention and Tobacco Control Act in 2009, the US company Kretek International, the parent company behind Djarum clove cigarettes, in anticipation of the clove cigarette ban, experimented with new products, including slim clove cigars that would circumvent the ban. Sales of clove cigars rose rapidly after the clove cigarette ban, increasing dramatically between 2009 and 2012 [16]. It was also widely recognized that the 2009 Act aimed to protect US menthol tobacco product manufacturers limiting the health protective effects [57, 58]. What we see in these cases is a confluence of factors that weaken the legislation while at the same time advocates either miss or are unable to address technical limitations of the legislation. What this study demonstrates is that despite the factors that weaken legislation, the presence and persistence of NGOs and other health advocates can keep the issue on the agenda for future reform.

Both experiences represent an important lesson for health policy makers and should be internalized in future regulatory efforts to keep youth from initiating the use of tobacco products.

## Abbreviations

NGO: Non-governmental organization
WHO FCTC: Framework Convention on Tobacco Control

## Footnotes

1 Physicians for a Smoke Free Canada was established in 1985 (http://www.smoke-free.ca/eng_home/pschome_about.htm), the Canadian Cancer Society has been in existence since 1938 (http://www.cancer.ca/en/about-us/fighting-since-1938/?region=qc) and the Quebec Coalition for Tobacco Control was formed in 1996 (http://www.cqct.qc.ca/qui/qui.asp) .
